# Responsibility for chemical exposures: perspectives from small beauty salons and auto shops in southern metropolitan Tucson

**DOI:** 10.1186/s12889-021-10336-4

**Published:** 2021-02-02

**Authors:** Amanda A. Lee, Maia Ingram, Carolina Quijada, Andres Yubeta, Imelda Cortez, Nathan Lothrop, Paloma Beamer

**Affiliations:** 1 Mel and Enid Zuckerman College of Public Health, University of Arizona, Tucson, AZ USA; 2 School of Anthropology, University of Arizona, Tucson, AZ USA; 3 El Rio Health, Tucson, AZ USA; 4 Sonora Environmental Research Institute, Inc., Tucson, AZ USA

**Keywords:** Occupational health, Health inequities, Chemical exposures, Responsibility, Environmental justice, Health policies, United States

## Abstract

**Background:**

Throughout the United States, low-wage, minority workers are disproportionately affected by occupational illnesses and injuries. Chronic exposure to hazardous chemicals at work can lead to serious illnesses, contributing to health inequities. In this article, we expand on theories of ‘responsibilization’ in an occupational health context to reveal how responsibilities for workplace chemical exposures are negotiated by workers and owners in Latinx-owned small businesses.

**Methods:**

We conducted semi-structured interviews with a total of 22 workers and owners in auto repair shops and beauty salons – two high-risk industries – in Southern Metropolitan Tucson. Participants were asked about their insights into workplace chemical exposures and health. A qualitative analysis team with representation from all study partner organizations collectively coded and reviewed the interview data in QSR International’s NVivo 11 and identified overarching themes across the interviews.

**Results:**

We identified three primary themes: 1) ambivalence toward risks in the workplace; 2) shifting responsibilities for exposure protection at work; and 3) reflections on the system behind chemical exposure risks. Participants discussed the complexities that small businesses face in reducing chemical exposures.

**Conclusions:**

Through our analysis of the interviews, we examine how neoliberal occupational and environmental policies funnel responsibility for controlling chemical exposures down to individuals in small businesses with limited resources, obscuring the power structures that maintain environmental health injustices. We conclude with a call for upstream policy changes that more effectively regulate and hold accountable the manufacturers of chemical products used daily by small business workers.

**Supplementary Information:**

The online version contains supplementary material available at 10.1186/s12889-021-10336-4.

## Background

Throughout the United States (US), low-wage, minority workers are disproportionately affected by occupational illnesses and injuries, and many of these are Latinx and immigrant workers [1, 2]. Chronic exposure to toxic chemicals at work can lead to serious illnesses such as asthma, cancer, neurodegenerative diseases, and reproductive health issues [3]. These morbidities contribute to existing health inequities due to workers’ limited access to affordable, quality medical care [4]. Our study seeks to answer: How is responsibility for mitigating workplace chemical exposures distributed among different occupational health actors, such as small business owners and workers, product manufacturers, and policy makers? We explore how owners and workers in Latinx-owned small businesses respond to conflicting responsibilities and negotiate the health risks of using products with chemical ingredients that they have little to no control over.

Latinx workers compose a significant portion of the low-wage labor market and are at greater risk of experiencing occupational health inequities than nonminority workers [5]. They are more likely to be employed in high-risk occupations and are exposed to heat, pesticides, hazardous chemicals, cleaning agents, and other physical hazards [6]. Education levels, economic class, race/ethnicity, job skills, language barriers, and documentation status are among the dimensions shaping the composition of workers in low-wage jobs associated with greater levels of risk and physical strain [7]. Although reliable data about the racial/ethnic composition of workers is inconsistent [1], especially in cases involving undocumented labor [5], an AFL-CIO report showed that Latinx workers experience an 18% higher job fatality rate compared to the overall workforce [2].

Low-wage, Latinx workers in high-risk industries – such as beauty and automotive industries – are increasingly vulnerable to occupational exposures from under-regulated chemical ingredients and the rolling back of social welfare programs, such as Medicaid and disability [8, 9]. Exact chemicals and dosages that workers are exposed to are poorly understood. A single workplace can be a site of multiple exposures to potentially toxic agents [1]. Volatile organic chemicals (VOCs) with known health effects have been documented in nail salons (e.g., toluene and methyl methacrylate [10]) and auto shops (e.g., benzene and xylenes [11]). These potential hazards on the job are exacerbated by temporary or informal work contracts, which often cause immigrant workers to go without health care, and many do not understand or are not offered workers’ compensation benefits in cases of workplace injuries. This may be compounded by fear of unemployment or deportation, making Latinx workers more reluctant to speak up about occupational hazards [6, 12]. Many of these challenges are also shared by Latinx small business owners and managers.

Lower incomes, limited opportunity for job growth, and workplace discrimination prompt some Latinx people to go into business for themselves. The number of Hispanic-owned businesses grew 46.3% from 2007 to 2012 [13]. However, Latinx businesses must overcome many barriers in order to achieve long term success, including labor market discrimination and difficulties accessing loans or other startup capital [14]. Many of these businesses are located in minority communities (with one study showing 58% of small urban businesses serving primarily minority clientele being minority-owned) where they can provide services to customers of the same race/ethnicity while avoiding discrimination and hostility in other areas [15]. Stress brought on by feelings of discrimination can be diminished through successful incorporation of their business into the community [16]. However, certain processes from small businesses, particularly auto repair and beauty shops, may also increase air pollution concentrations in their neighborhoods, increasing environmental health disparities. Chemical exposures, which occur frequently in auto shops and beauty salons, are a leading cause of occupational injury, but many Latinx-owned small businesses face additional barriers in implementing workplace safety measures [6].

In the U.S., greater than 55% of the private workforce is employed in small businesses (< 100 employees) [17]. Owners of small businesses have a more difficult time meeting occupational health and safety measures due to the limited personnel and economic resources available for implementation. A lack of workplace regulations, in turn, makes employees responsible for their personal wellbeing and leaves health and safety problems to be solved after they occur [18]. An employer’s willingness and ability to address a hazard contributes to the severity of risk from the hazard, which may lead to increased negative health effects for workers [7]. Additionally, small businesses are less likely to employ industrial hygiene consultants or have medical surveillance programs and the large number of these businesses exceeds the capacity of government programs to provide safety assistance [17]. For our study, we focus on small beauty salons and auto repair shops in southern Arizona. One in every 200 jobs is in a beauty salon or auto repair shop (excluding contract workers) and more than half of salon workers (53%) and three-fourths of auto shop workers (77%) are employed in small businesses with fewer than 20 employees [19, 20]. Health risks from chemical exposures are widely documented in nail salons and auto body shops [11, 21–23]. Our project expands this research into *beauty* salons and auto *repair* shops, which are more numerous and often provide mixed services (in addition to nail care and auto body work, respectively).

### Theoretical framework: neoliberalism, structural vulnerability, and responsibility

Social theories about neoliberalism and structural vulnerability inform our understandings about the ways in which occupational health policies influence chemical exposures in small businesses. Neoliberalism is “a theory of political economic practices that proposes human well-being can best be advanced by liberating individual entrepreneurial freedoms and skills within an institutional framework characterized by strong private property rights, free markets, and free trade” [24]. Social scientists, such as geographer David Harvey, have criticized neoliberal ideology because it overemphasizes individual agency and decision-making, while deregulating profit-driven industries and multinational corporations (2005). Coupled with neoliberalism are structural vulnerabilities, the historical, political, and social constraints on individuals that limit their agency over their health and economic circumstances (Farmer 2004). In occupational health, neoliberal policies increase the structural vulnerability of workers in high-risk industries by reducing their ability to control chemical exposures while simultaneously increasing their responsibility to protect themselves from those exposures.

Relations of responsibility between state and subject actors “are constantly being asserted, contested, and redrawn” [25]. An individual’s ambivalence toward risk responsibility is bound up with their perception of agency and relationship to the institutions regulating responsibility. As Melanie Pescud et al. (2015) demonstrated, responsibility for workplace health is often ambiguous between employers and workers, especially in small businesses where employers are reluctant to cross boundaries between occupational safety and personal autonomy over health decisions. Employers express competing ideas that they are both responsible for the occupational safety of their workers, but also that they cannot dictate workers’ lifestyle choices [26]. In this article, we expand on theories of ‘responsibilization’ – meaning the decentralization of responsibility to individual actors [27] – in an occupational health context to elucidate how workplace safety responsibilities are negotiated among people in small businesses and larger state or corporate actors. Though responsibility underpins social relations of care [28], workers are subject to ideals of neoliberal responsibilization that restrict their ability to be accountable for reducing environmental risks for themselves, as well as their coworkers.

Neoliberal policies limit regulations on product manufacturers and increase the burdens on individuals to protect themselves and take care of their own health, contributing to the structural vulnerability of racialized workers in particular [9, 29]. In response, workers in high-risk industries may construct ambivalent understandings about the dangers of their professions that both internalize and oppose risk responsibility. Through these constructions, small business owners and workers may reiterate neoliberal ideas about individual responsibility for workplace safety, as well as shift responsibility among different occupational health actors, from workers in the shop to industry and government entities. Neoliberal theory provides a lens for understanding how responsibility for reducing chemical exposure is distributed among these actors.

### Occupational health policies in the United States

Recent and ongoing cutbacks to US regulatory institutions like the Environmental Protection Agency (EPA) and the Department of Labor (DOL), which houses the Occupational Safety and Health Administration (OSHA), as well as limited occupational health resources for small businesses, perpetuate worker vulnerability to chemical exposures [12, 30]. In tandem with this, the Toxic Substances Control Act of 1976 (TSCA), which regulates chemicals with “unreasonable risk” to human health or the environment, faces significant barriers to implementation. Early on, TSCA encountered challenges as thousands of existing chemicals were already in commerce, leaving regulators with little influence over their control. Congress also made it more difficult for the EPA to have influence on safer chemicals for the future by drastically restricting enforcement of “blanket testing requirements” for all new chemicals within TSCA. The legal burden to prove that a chemical is hazardous to health was placed on the prosecutor (i.e. the EPA) and not the manufacturer. Though TSCA was recently updated, many issues of enforcement persist due to lobbying efforts from manufacturing companies. These neoliberal restrictions on government regulations and the absence of laws requiring the provision of minimal toxicity information cause uncertainties surrounding safety data provided by manufacturers [31].

Labor policies such as the Immigration Reform and Control Act of 1986, the Illegal Immigration Reform and Immigrant Responsibility Act of 1996, and Personal Responsibility and Work Opportunity Reconciliation Act of 1996 have incentivized employers to subcontract workers – given that OSHA does not require employers to provide protection from workplace hazards to independent contractors – while also restricting their access to health services [5, 8]. These large-scale policies influence how Latinx-owned small businesses operate under the radar in comparison to larger workplaces. Many workers in small businesses, particularly beauty salon workers, are self-employed and either are hired as independent contractors or rent space from the business owners. There is little information collected about non-traditional workers [17], but these workers have high job fatality rates and limited safety resources [2]. Often small businesses also hire family members, blurring the lines between employers and employees. These policies complicate safety enforcement in shops and produce uneven access to hazard protection resources provided by OSHA and OSHA-approved State Plans. Attention to policies, workplace health regulations, safety trainings, and federal protections targeting industries known for employing immigrant workers in high-risk jobs may help reduce occupational injuries and fatalities [32].

OSHA is a federal agency established to ensure “safe and healthful conditions for working men and women by setting and enforcing standards and providing training, outreach, education and compliance assistance” [33]. It institutes and enforces safety regulations for businesses, issuing citations and fines to non-compliant employers. On-site consultation visits can be set up to have OSHA consultants identify and provide advice on mitigating potential workplace hazards for small businesses. Though their website emphasizes that the consultants do not report safety violations, it also states that the OSHA enforcement office will become involved if businesses fail to follow consultant recommendations, causing many small businesses to avoid setting up consultations [34]. While these efforts were intended to protect workers from occupational hazards, a combination of too few OSHA inspectors, low penalties, and historically limited funding make these resources inadequate to address the problems that could ensure a safe workplace [2].

**Fig. 1:**
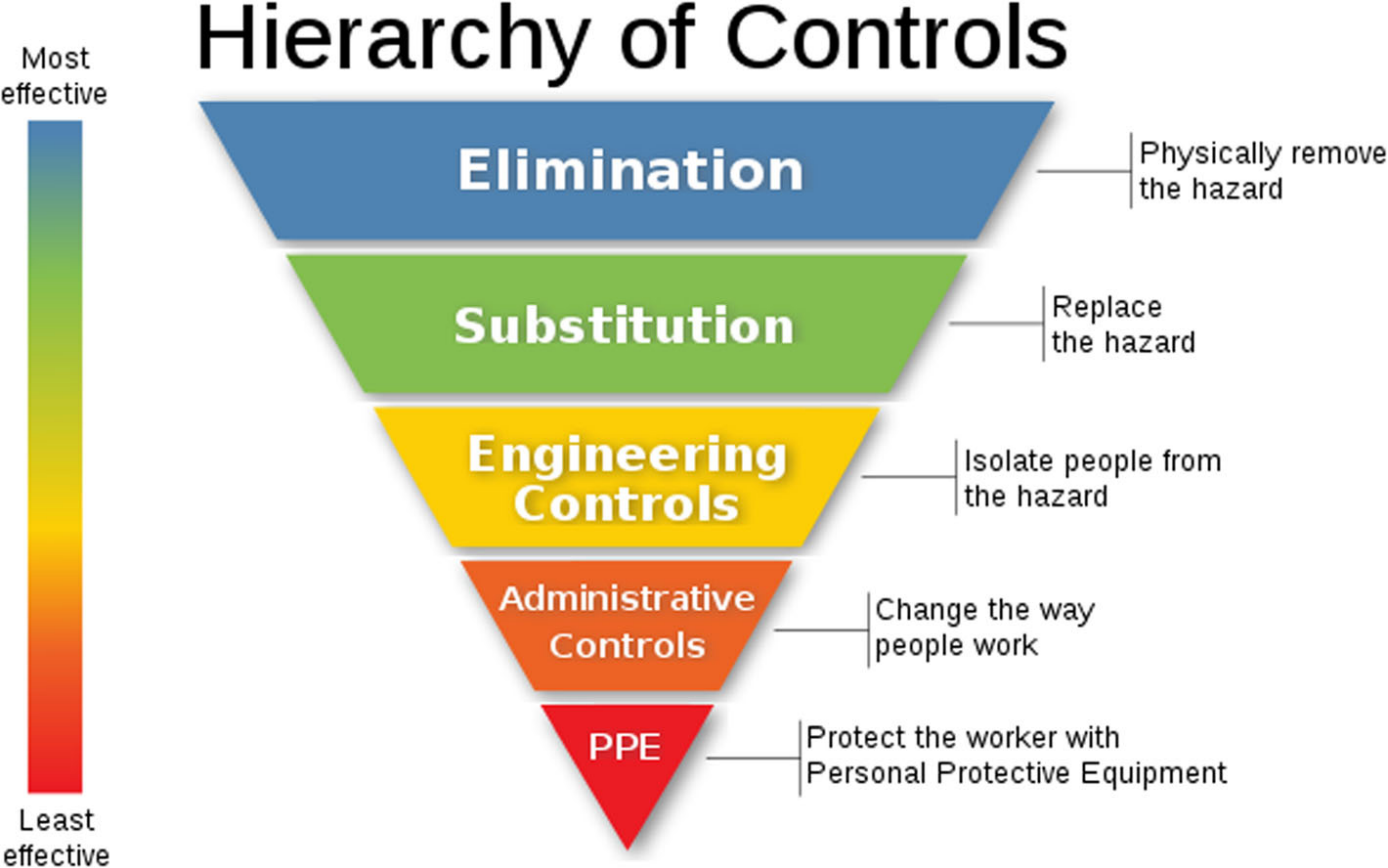
The National Institute for Occupational Safety and Health’s Hierarchy of Controls. Source: U.S. National Institute for Occupational Safety and Health [35]

As part of its official hazard reduction recommendations, OSHA promotes the Hierarchy of Controls framework to prioritize methods for controlling and reducing hazards in workplace (Fig. 1). While not by design, in small businesses, this framework directs responsibility for managing risk onto individual business owners and workers. The most effective methods in the hierarchy are to physically eliminate or substitute a hazard, an option that is often not possible when using products with toxic chemicals in high-risk industries due to limited choices and confusing information about how ‘safe’ a product is. Small businesses lack sufficient economic resources for making often expensive engineering changes such as installing ventilation systems and purchasing new equipment [30]. Even the less effective options on the hierarchy, which focus on worker behaviors, can be difficult to implement because they may interrupt workflow and they depend on access to personal protective equipment (PPE).

Current policies around occupational and environmental hazards, such as those described in this section, enforce who has or does not have access to governmental protections and orients responsibility for risk toward individuals. Changing behaviors at work and educating workers about potential hazards are the target of many occupational health efforts, like the Hierarchy of Controls. Anthropologist Sarah Horton demonstrates how intersecting labor, welfare, immigration, and health policies shape the lives of farmworkers suffering from heat exposure. These policies produce structural vulnerability to occupational health risks for the workers, while hazard reduction campaigns tend to emphasize the importance of individual decision-making and education to reduce ‘risky’ behaviors. Experts advocate for workers to learn about the symptoms of heat illness and to take breaks when they experience symptoms. Horton describes this as “misplaced autonomy” because the knowledge about what causes heat exhaustion does little to help workers forced to continue working under hazardous conditions [36]. This funneling of responsibility to individual workers for their wellbeing, and not toward the larger structures producing risk, overlooks the already-reduced agency of low-wage workers, who need a paycheck to support themselves and their families and may not have the option of changing to less hazardous occupations.

The objective of our research is to identify ways that beauty salons and auto shops in Tucson, Arizona can reduce worker chemical exposures in the workplace. These small businesses are sites where the effects of structural responses to environmental hazards, such as chemical safety standards and occupational regulations, can be observed in how they impact workers’ daily lives. Through this project, we draw on scholarship about responsibilization to argue that individualistic occupational safety efforts often inadequately address the root sources of exposures to environmental health risks.

## Methods

### Study setting & context

**Fig. 2:**
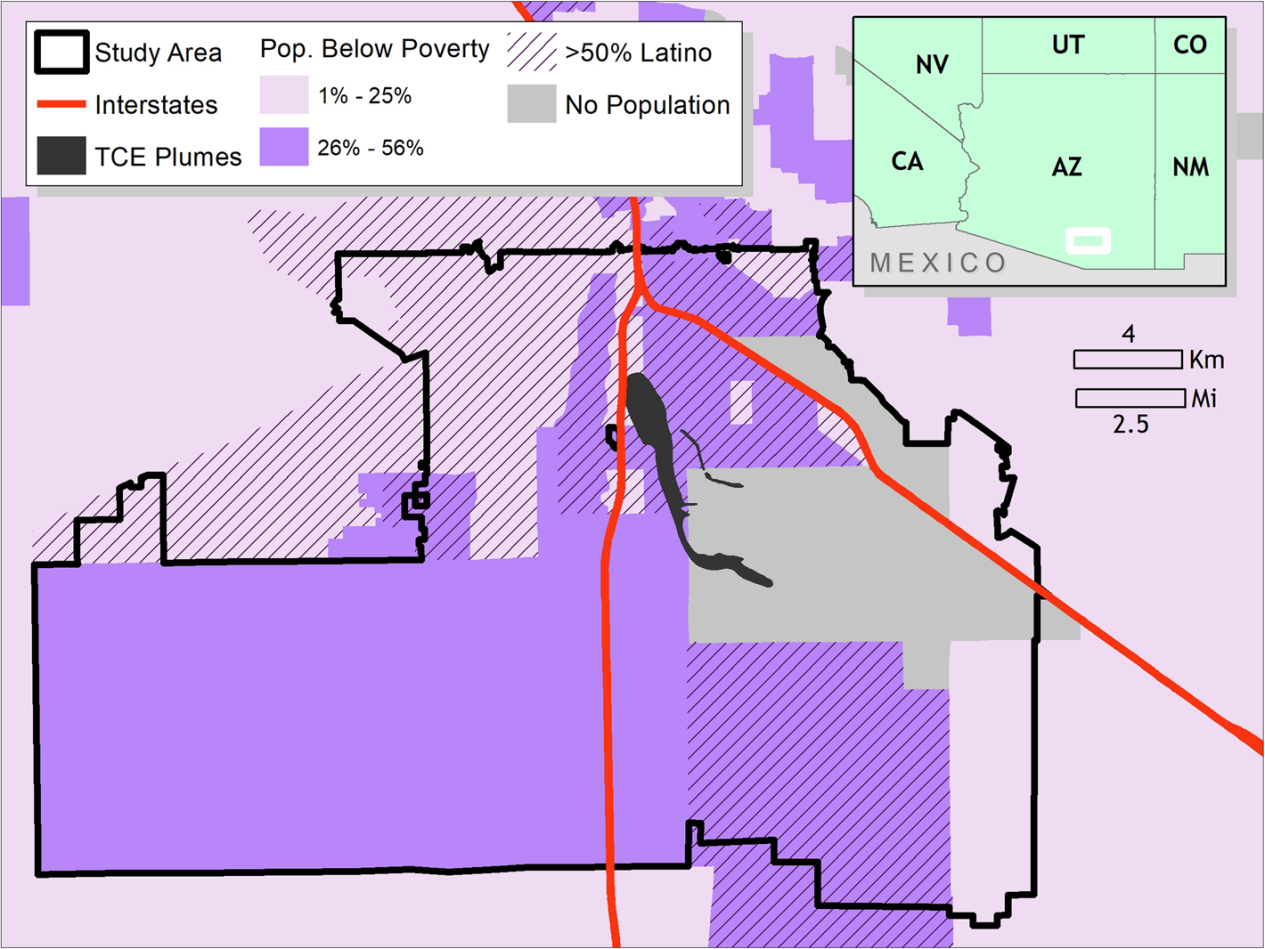
Map of study area, population characteristics, interstates, and TCE plumes from the TIAA Superfund Site. Sources: U.S. Census Bureau, Pima County GIS, and the City of Tucson [37–39]. Note: This original map was generated by NL for this paper using ArcGIS 10.6.1 (ESRI, Redlands, CA)

Understanding responsibility for the reduction of workers’ chemical exposure has important implications for small businesses in southern metropolitan Tucson, a primarily Latinx community facing significant health and economic inequities. A legacy of environmental racism has contributed to increased pollutants in the air and water of low-income, predominately Latinx neighborhoods, increasing overall cumulative risk for workers who both live and work in these areas. Located nearby are major highways that contribute to air pollution and the Tucson International Airport Area (TIAA) Superfund Site where carcinogenic chemicals such as trichloroethylene (TCE) were dumped into groundwater during industrial activities from 1951 to 1977 (Fig. 2). Reports about escalating health effects from communities affected by the TCE plumes were not acknowledged or acted upon by authoritative political figures until long after community members started voicing their concerns [40].

Our study is part of the first phase of an R01 funded research project, *Tu Trabajo no te Debe Dañar: Reduction of Hazardous Exposures in Small Businesses through a Community Health Worker Intervention*. The overall purpose of the parent study is to implement and evaluate a *promotora*-led industrial hygiene intervention developed by the Sonora Environmental Research Institute (SERI) to decrease exposures in small businesses (< 20 employees) [41]. SERI’s intervention provided the basis for a research partnership with the University of Arizona and El Rio Community Health Center. In this community-engaged study, staff from each of the organizations make up the members of the research team who share responsibility for all aspects of the research, from identifying the health issue of concern to data analysis and dissemination of results [42]. This collaborative and participatory approach to research increases the quality and relevance of research questions and increases the potential that the results will bring about in solutions that are feasible and acceptable to small businesses and their employees [43]. Our ongoing research aims to identify and carry out potential opportunities for reducing worker chemical exposures in small businesses while addressing the social, physical, and economic barriers for affordable safety resources.

In the summer of 2018, our team conducted a study with the goal of gathering data on chemical exposures and perceptions of health risks in small auto and beauty shops. In these high-risk, service-industry businesses, workers are regularly exposed to chemicals from the products they use during shop activities. Auto shops frequently use degreasers, paints, and other chemical products, while beauty salons may work with dyes, relaxers, hairsprays, and nail polishes. We monitored worker activities to identify when the greatest exposures occurred and are using these quantitative findings to inform our ongoing exposure-reduction intervention.

As part of this research, we interviewed 22 study participants about chemicals and health in the workplace. In this article, we discuss three main themes that we found throughout the interviews to ultimately demonstrate how neoliberal occupational health policies obscure larger structures of power that perpetuate an increased burden of chemical exposures in low-income communities of color.

### Data collection

The collaborative research team developed a semi-structured research guide to interview small business owners, managers, and workers about their insights into workplace chemical exposures and health (see Additional file 1: Interview Guide). The guide was structured to orient the respondents in a reflection of their overall perceptions of health in general (e.g., what does health mean to you?) and then to focus more specifically on aspects of the work environment related to health (e.g., what affects your health at work?). The guide then asked questions specific to potential work exposures and included probes to identify the measures that workers and owners took to protect create a safe workplace. We also specifically asked who was responsible for safety at their place at work. We piloted the questions with an auto body shop and beauty shop owner. The pilot study revealed an issue with translating the word “safety” literally into Spanish as *“seguridad”* or personal safety. Ultimately, we did not use the word “safety” in the Spanish version of the interview guide, instead conducting a functionalist translation that focused on the intended concepts of the focus group [44].

In the first phase of the study, SERI *promotoras* visited numerous shops and salons, each with unique characteristics. Some were small and family operated, while others were larger, with high employee turnover; all reported having ten or fewer employees. There were auto shops that were primarily open to the natural elements (e.g., the desert heat), while others had up-to-date, enclosed ventilation systems for painting cars. The shops had their own specialties, such as car body work or mechanical repairs. The beauty salons also varied in structure, some with shared product lines and others with individual booths rented out by the shop owner. Their clientele varied in age range, race/ethnicity, and economic status, which influenced the types of services they provided. Whether or not business owners owned or rented the building or property also varied from workplace to workplace.

We recruited workers and owners for the interviews from businesses that had agreed to be part of the larger intervention study. El Rio collaborators contacted individuals who agreed to participate in the interviews to schedule an interview. When given a choice of where they would prefer to complete the interviews, most participants chose their workplace. All participants were over 18. Written consent was obtained, and participants were interviewed in their choice of English or Spanish. All Spanish quotes have been translated into English, alongside their original language. Interviews typically lasted 20–30 min. All procedures were approved by the University of Arizona Institutional Review Board.

### Data analysis

Each interview was transcribed in their respective language by study team members. The interviews were all coded in QSR International’s NVivo 11 using deductive codes derived from the Hierarchy of Controls and the Socio-Ecological Model (SEM). The hierarchy controls provided a framework for assessing perceptions of responsibility for risk, while the SEM focused on the multiple and converging individual, social, and economic factors that influence those perspectives [43]. As a group, we developed the codebook together in English and Spanish so that we each had a shared understanding of what content fit under which codes. The codes included definitions for each level of the Hierarchy of Controls and the SEM, tailored to address the specific context of this study. For example, the “Elimination / Eliminación” code included content “about eliminating or removing an environmental hazard in the workplace / sobre la eliminación de un peligro ambiental en el lugar de trabajo.” Two researchers were assigned to code each interview and a qualitative analysis team with representation from each study partner organization collectively reviewed the coded data for every code over the course of several meetings. Using modified grounded theory techniques [45], we reached group consensus to identify topics across the interviews that emerged from in-depth discussion of each code. These topics were grouped into three overarching themes: 1) ambivalence toward risks in the workplace; 2) shifting responsibilities for exposure protection at work; and 3) reflections on the system behind chemical exposure risks. The themes and quotes shared in this article were agreed upon by the team, who are all co-authors, after reflection about their salience throughout a significant portion of interviews and relevance to the broad experiences if study participants. Though these interviews are analyzed here together, we discuss differences in how these themes were addressed among workers versus owners and in auto shops versus beauty salons when applicable.

## Results

We conducted interviews with a total of 14 owners/managers and 8 workers from 16 shops (Table 1). Eleven of the interviews were with workers in beauty salons, who were all women, and nine were in auto shops, where interviewees were almost all men. Most interview participants had worked in their respective industries for at least ten years and all but one of the participants identified as Latinx.

**Table 1:** Interview Participant Characteristics

	Beauty Salons N (%)	Auto Shops N (%)
Ethnicity		
Latinx	11 (100)	10 (91)
Non-Latinx	0 (0)	1 (9)
Gender		
Female	11 (100)	1 (9)
Male	0 (0)	10 (91)
Shop Role		
Owner/Manager	7 (64)	7 (64)
Worker	4 (36)	4 (36)
Age (mean, range)	48.1, 35–65	41.6, 24–56
Years Working in the Field (mean, range)	21.3, 8–40	22.3, 1–45

### “It comes with the territory”: ambivalence toward risks

The people in our study expressed ambivalence about the risks of their work. Across the interviews, managers and workers in small auto and beauty shops expressed an awareness that the products they used contain potentially harmful chemicals, but also resigned themselves to the inevitability of exposure. They made evaluations about the ingredients of products used in their shops that ranged from concern about how “strong” the chemicals were to doubt about the health risks. A beauty shop manager voiced concerns about the effects that the chemicals might have on her health, especially from hair treatments like perms, but explained that she could not only cut hair *“porque es un salón de belleza”* (because it’s a beauty salon). Others were resigned to the prospect of future health issues from the chemicals. Several managers told us that they attempted to buy the “safest” products, which they sometimes based on the smell of products or the “organic” or “natural” labels.

On the other hand, many workers and managers told us that they were not very worried about the chemicals in their shops. One beauty shop worker commented: *“A lo mejor es un factor de riesgo, pero pues igual como te digo hay gente que no tiene nada que ver y se enferman de lo mismo pues.”* (Maybe it’s [working in the beauty shop is] a risk factor, but like I said, there are people who have nothing to do with this work and get sick anyway.) A few people reasoned that there are health risks at any job, casting doubt on feelings that their occupation might be more dangerous than any other. An auto shop manager dismissed the risks when asked if he was worried about workplace exposures, saying *“todo en exceso siempre es malo”* (everything in excess is always bad).

Ambivalent perspectives about chemical exposure in the workplace were influenced by workers’ individual experiences working in the auto and beauty industries. An auto shop employee in his thirties explained that he had not thought about his daily exposure before participating in our study:

*I never considered how much that [exposure to chemicals] could be affecting my health. And I guess the other side of that is that I’ve been doing this for so long and I haven’t really had any adverse effects, so I figure it’s safe.*

His lack of noticeable health effects led him to feel secure about the products he used on the job. Several people expressed a reliance on their own years of experience and expertise in their fields to know whether something was safe or not: *“I’ve been here more than 27 years, so whatever it [chemical] is that shouldn’t be, too late. I’m immune to it or whatever.”* Participants frequently expressed this sentiment.

Alternatively, others who had previous issues with their health were more worried. Two women working in the same beauty salon told us about going to see doctors for respiratory and allergy problems. One of the women was told by a doctor she had *“asma provocada por todo el químico que tu utilices”* (asthma caused by all of the chemicals you use) and was advised to stop working in the salon. She expressed to us that she felt she could not change careers after over twenty years and had to continue working in an industry that affected her health.

Both managers and workers negotiated their ‘exposed’ positions and limited resources in order to explain why they continued to work in high-risk occupations. As one auto shop worker described:

*They’re [the chemicals are] bad for us, you know, but I mean, we gotta work… And I know it’s gonna probably- it’s gonna harm me eventually… But, like I said, I’m just- it’s what I know how to do and I’m just stuck in it now.*

Health issues, many told us, were inevitable: *“in this line of work… it comes with the territory.”* Overall, people were aware of risks associated with working in a profession with daily exposure to potentially harmful chemicals. A beauty shop manager told us: *“Cuando tu estás trabajando con químicos sabes que te va afectar de alguna manera tarde o temprano.”* (When you’re working with chemicals, you know it will affect you somehow sooner or later). Despite this, many participants felt they had to continue working and relying on their own evaluations about safety in order to make a living. As managers and workers in small beauty and auto businesses with limited resources, the knowledge of risk was troubling, but something they had to contend with every day.

### “It’s for their own protection”: shifting responsibilities at work

When asked about responsibility, managers emphasized their role in maintaining a safe environment but shifted ultimate responsibility to reduce personal chemical exposures onto the workers through their framing of individual decision-making. Most interview participants expressed that they felt responsible for their own utilization of protective personal protective equipment (PPE) and for their knowledge about the chemicals in certain products.

Business owners and managers discussed workers’ “choices” to wear PPE, to attend safety trainings, or to work in their shops at all. Many managers in auto shops emphasized that they provided PPE such as gloves and masks, but that it was up to the workers to wear it. They reported that they tried to encourage or enforce workers to wear PPE. As one manager explained: *“I supply the gloves for them. I supply the mask, you know. I catch them sometimes not wearing them. And I get on their case. Cause they’re supposed to wear them. It’s for their own protection.”* This manager repeated what is “supposed to” be done by the workers several times, creating distance between him as a manager from taking responsibility for the individual actions of his workers.

Beauty salon owners differed from auto shop owners because the workers in their salons were usually self-employed and therefore responsible for providing their own PPE and beauty products. This individual worker responsibility to use less hazardous products is particularly important because in many small businesses like these, there are just a few employees, meaning that owners and managers often work alongside their employees, exposing them to the same chemical risks and need for protection. Despite this, several managers emphasized that *“they [the employees] do their own thing”* or *“yo les digo úsenlos”* (I tell them to use them [PPE]), rarely reflecting on their own PPE use in our interviews.

At the same time, workers often placed blame on themselves if they did not wear PPE. One worker commented, *“I’m trying to get my job done so I don’t think about putting on eyeglasses.”* Constraints on personal ‘choices’ to take extra protections ranged from workflow interruptions, costs, and client preferences. They explained that it was *“más fácil manejar todo sin guantes”* (easier to handle everything without gloves). PPE was cumbersome and uncomfortable to wear throughout the day. These reflections focused on PPE use, the least effective control, rather than more effective measures such as eliminating chemical hazards or installing ventilation equipment, which were seldom mentioned by workers or managers. Some beauty shop workers made comments about trying to substitute with products that they believed were safer. One woman emphasized: *“trato de usar lo más natural que se pueda”* (I try to use as natural [products] as I can). But interviewees said these options were limited due to costs and availability. Furthermore, many people mentioned that work was not a site to worry about health, but to be productive. When asked specifically about who was responsible for their health and safety at work, many workers answered *“yo mismo”* (myself).

In beauty shops, manager and worker risk responsibilities were not only concerned with employee protections, but also with client protections. Sometimes, concerns for clients’ needs superseded concerns for their own health. One salon manager told us that she agreed to participate in the study because she was worried about the children who came into the shop *“nadando en químicos”* (swimming in chemicals). She mentioned opening doors in these cases when kids were around to increase airflow. Others brought up being worried about older clients’ exposure to chemicals, but these concerns were dichotomized with needing to satisfy the clients’ desires. For example, a manager explained that people wanted dyes that would cover grey hairs the best, limiting her product options. Client preferences took precedence over using “natural” chemicals because, in the end, *“vienen por un servicio y me pagan por eso”* (they come in for a service and they pay me for that). These shifting responsibilities further complicate efforts to reduce exposures in workplaces.

### “It’s really their responsibility”: reflections on the system

In addition to feeling individually responsible for their own safety, many participants working in these industries also reflected on systemic issues contributing to their exposures at work. These included inadequate access to safety information and an inability to change the ingredients put into their products or to afford upgraded safety equipment. These larger structural problems were beyond their power to easily change as small businesses: *“there’s nothing new that we can do to change it [worker safety] ‘cause we’re already- we’re a small business, we’re always looking at ways to save.”* Instead, some people put trust into manufacturers to follow chemical regulations and make the safest products possible.

When asked about barriers to reducing chemicals in the workplace, a manager in an auto shop told us: *“las barreras seria la falta de información”* (the barriers would be lack of information). Multiple people expressed that they wanted more education and training about the product ingredients and how to reduce their exposures. *“Que les digan en la escuela”* (Tell them at school), one beautician proposed, while reflecting on what she wished she had known about the health effects going into the industry as a beauty school student. Throughout the study, people used a variety of words to describe chemicals, including: *“green,” “tóxicos,” “bio-friendly,” “hazardous,” “orgánicos,” “ammonia-free,”* and so on. Interviewees did not feel confident in their ability to place certain chemicals on the continuum of safety, adding to the confusing information about risks. Several people mentioned that they tried to read the product labels and accompanying Safety Data Sheets if they had them, but that they still felt unsure about what this information meant for their health: *“Muchos términos vienen en términos científicos que si yo leo la palabra ni sé que significa, ni se ni lo que es.”* (Many terms come in scientific terms that if I read the word, I don’t know what it means, I don’t even know what it is.)

Some managers and workers explicitly pointed to the responsibilities of the companies selling them products: *“it’s up to the manufacturers to use less hazardous materials.”* These responses critiqued the lack of transparency about safety from sellers and their preoccupation with making a profit over the impacts of dangerous chemicals on the bodies of workers like themselves. Some people questioned the capitalist ethics of companies profiting from their exposures. A white, non-Latinx auto shop worker emphasized his feeling that exposure education was ultimately up to the product manufacturers:

*It’s all about making a buck with everything, but I believe that it’s down to responsibility of the company that [is] selling any of their products to anyone because they- it’s really their responsibility to provide information, you know? Even though it’s just scribbled on the back of a tin or something. If you’re supplying a company, it should be down to them to provide the information to the company for the employees to know what they’re working with.*

He questioned who is “really” responsible, deflecting responsibility away from individuals in the shop and re-positioning it toward larger structures. Though he acknowledges that companies provide some information, he indicates that this is not enough. Another worker even felt that automotive safety certification courses were a “money grab.” However, some other workers and managers contradicted this distrust, saying that they believed the companies were already making the products as safe as possible. One worker reasoned that the chemicals were *“getting better because they don’t work as good as they used to,”* while others remarked on the improved scent of products and the quality of some brands over others.

Judgements about how risky the work is, who is responsible for controlling exposures, and the importance of safety information and regulations varied among participants. When asked about potential solutions, a beauty shop manager in her fifties with 25 years of experience in the field proposed advocating for *“campañas a las compañías, para que reduzcan un poco los riesgos o los ingredientes muy nocivos para la salud.”* (Petitioning the companies so that they reduce the risks a little bit or the ingredients that are very harmful to health). To her, this would be an important step, not only for workers, but also for the larger community that comes into these shops.

## Discussion

In this study, we demonstrate how individualistic policies place the onus of chemical exposure reduction on small businesses, hiding the larger corporate and state power structures that maintain health inequities and environmental injustices. Small business workers and owners responded to questions about workplace safety by emphasizing personal responsibility for self-protection measures, while also acknowledging that they had insufficient information about or a lack of control over the types of chemicals in their shops. Interview participants expressed conflicting views about who controls chemical hazards at work, often shifting responsibility between workers and employees, management, and product manufacturers. Personal evaluations of their overall health despite daily chemical exposures at work allowed people to rationalize the risks of their occupations. Others acknowledged and worried about the effects of exposures on their health, but also felt powerless over the chemicals that they had to use. In beauty salons, many felt that their safety was secondary to the preferences of their clients to use products that work better to achieve a desired look. These ambiguous stances toward responsibility for workplace safety left shop workers and managers to make sense of the risks in their line of work and negotiate their limited roles in reducing those risks. Often those negotiations placed greater pressure on individuals to make complex safety decisions without adequate resources or information. Institutional culpability for occupational health issues was simultaneously critiqued by workers and deflected away from companies manufacturing products with hazardous ingredients.

Our results reflect worker perspectives about chemicals and health that are influenced by the broader sociopolitical context disproportionately exposing low-wage, minority workers in Tucson to under-regulated chemicals and individualistic occupational health policies. We reveal the complexities that small businesses face in reducing chemical exposures. Workers in these high-risk industries bear the health consequences of mainstream neoliberal responsibilization that is too reliant on the individuals’ agency to purchase safe products, effectively wear PPE, and eliminate their own exposures. The Hierarchy of Controls promoted by OSHA sets a standard for safety approaches but neglects to acknowledge the hierarchy of agency between those who manufacture products and those who use them. Though employers are encouraged to follow the hierarchy framework, our interview participants demonstrated that the most effective hierarchy controls (eliminating/substituting hazards or installing engineering controls) are often impossible in small Latinx-owned businesses, where there is a dearth of financial resources, OSHA availability, and Spanish and plain language informational materials. These resources may be more difficult to access for contract and immigrant workers facing multiple forms of vulnerability. To apply a hierarchical framework for reducing workplace risks, one must look toward greater chemical regulations and corporate accountability to “eliminate” these hazards.

In an environment where people are exposed to hazardous chemicals both at home and at work, it is difficult to know what causes certain health issues, a point that is used by officials such as policymakers and manufacturers to avoid taking action to reduce environmental exposures [40, 46]. Rising cutbacks to regulatory institutions make it even more challenging to regulate and evaluate the chemical ingredients that large manufacturers add to products [12]. Many of the chemicals in auto and beauty products used daily in shops have not been tested for toxicity, and the onus is on regulators to prove that a chemical is hazardous before it can be removed from the market. Emphasis is placed on eliminating and preventing exposure at the level of individual shops, but the companies who generate the dangers in the first place are not held accountable. As anthropologist Catherine Hodge McCoid writes about the environmental factors that increase the risk of developing breast cancer, “breast cancer policy tends to be directed toward ‘fixing up’ the problem after it has occurred, rather than toward genuine environmental prevention.” She argues that this is due to “bottom-line” thinking that favors short-term profits for companies over addressing the long-term consequences of toxic chemicals on the environment and health outcomes [47]. More effort is placed into less effective, downstream policies instead of targeting the source of pollutants.

In an occupational health context, misplaced autonomy puts increased structural vulnerabilities onto individual workers who cannot easily change professions [36]. Environmental exposure to hazardous chemicals in poor, minority communities perpetuates health inequities, and neoliberal policies directed at worker health fail to address issues of environmental justice [48]. Our ongoing study seeks to provide workers with greater control over their occupational exposures through community-level education and resource development. Future directions of research might more closely examine differences between exposures in auto shops versus beauty salons and the role of gender in assessing risk responsibilities in small businesses. Additionally, more research is needed on the chemicals used in automotive and beauty products in order to inform and promote evidence-based, upstream policy changes for protecting worker health.

Though the research presented in this article occurred before the COVID-19 pandemic, the issues that workers shared have become even more pressing. Small businesses are reopening out of economic necessity, even in areas of widespread community transmission. Responsibility is being placed on small business to develop procedures for minimizing risk with almost no guidance. They must provide appropriate yet difficult to obtain PPE and enhance their cleaning and disinfectant procedures, increasing workers’ exposures to chemicals as they grapple with reducing exposures to the virus. Clear and consistent guidance and policies are desperately needed from federal, state, and local agencies to help reduce transmission of COVID-19, prevent additional unnecessary chemical exposures, and protect the health of low-income and minority workers. Ultimately, without a comprehensive and systemic commitment to worker health and safety that goes beyond individual behaviors, the public health and economic burdens of the pandemic will continue to disproportionately impact working class communities of color.

## Conclusions

In our study, we found that manufacturers’ role in chemical exposure risk in small, minority-owned businesses was diminished or obscured by a lack of adequate information about environmental risks; a sense of inevitability toward health repercussions caused by exposures; and unclear or ambivalent feelings of responsibility. Policies centered around worker- and shop-level safety education and individual ‘risky’ behavior changes overlook the realities of exposed workers. They gloss over corporate accountability for using misleading product labels and instructions. Federal, state, and local policies and protections for worker health should be critically examined in how they funnel responsibility for chemical hazards down to individuals instead of addressing the larger social forces that manufacture uneven exposures.

## Supplementary Information

**Additional file 1.** Interview Guide. Description: Questions and probes from the semi-structured interview guide developed by the authors for this study and grouped by Worker/Employee Questions and Owner/Manager Questions.

## Data Availability

To protect the anonymity of the participants, the datasets generated and analyzed during the study are not publicly available but are available from the corresponding author on reasonable request.

## Abbreviations

DOL: Department of Labor
EPA: Environmental Protection Agency
OSHA: Occupational Safety and Health Administration
PPE: Personal Protective Equipment
SEM: Socio-Ecological Model
SERI: Sonora Environmental Research Institute
TCE: Trichloroethylene
TIAA: Tucson International Airport Area
TSCA: Toxic Substances Control Act of 1976
US: United States
