# Supplementary material for: Responsibility for chemical exposures: perspectives from small beauty salons and auto shops in southern metropolitan Tucson
Source: BMC Public Health. 2021 Feb 2;21:271. doi: 10.1186/s12889-021-10336-4 (PMC7851808; doi:10.1186/s12889-021-10336-4)
Supplement: Supplementary file 1 — Additional file 1. Interview Guide. Description: Questions and probes from the semi-structured interview guide developed by the authors for this study and grouped by Worker/Employee Questions and Owner/Manager Questions. [file 12889_2021_10336_MOESM1_ESM.pdf]

<u>Interview Guide</u>

Worker/Employee Questions:

1. What does "health" mean to you?
2. For you, what affects your health at work?
    a. *Probe: Do you have any health concerns related to your work or your workplace?*
    b. *Probe: Do you know anyone who has gotten sick from work or exposures at work?*
3. What concerns about safety at work do you have?
4. Do you use chemicals at work?
    a. *Probe: For <u>beauty</u> sector shops: What about sprays, hair dye, bleach, perm/straightener, nail polish, etc.?*
    b. *Probe: For <u>auto</u> sector shops: What about sprays, degreasers, solvents, cleaners, paints, etc.?*
    c. *Probe: Do you worry about inhaling chemicals or getting them on your skin?*
    d. *Probe: How do you learn about working with chemicals?*
5. Do you feel like you have sufficient knowledge to work with these chemicals safely?
6. What personal protective equipment do you use at work? And who provides it?
7. How do you get trained to work with chemicals/products safely?
    a. *Probe: When do you get trained: at hire, periodically, annually, etc.?*
    b. *Probe: Who provides the training?*
    c. *Probe: How has training changed your behavior about health and safety?*
    d. *Probe: Has your health or health condition (yours, family, etc.) influenced your behavior about health and safety at work?*
8. Who is responsible for safety at your place of work?
    a. *Probe: Please give an example of how [you/owner/manager] do this?*
    b. *Probe: Who has the greatest influence on safety?*
    c. *Probe: Does your supervisor/owner talk about safety?*
    d. *Probe: Do workers talk to each other about safety? (Probe: Gender?)*
    e. *Probe: Who do you trust for advice on safely using chemicals at work?*
9. How does the amount of work you have influence your safety at work?
    a. *Probe: Work pace?*
    b. *Probe: Taking care of your health?*

Owner/Manager Questions:

1. What does health or being healthy mean to you?
2. How does your business or workplace support your employees' health?
3. What concerns do you have about health and safety at your business?
4. Do you use chemicals at work?
    a. *Probe: For <u>beauty</u> sector shops: What about solvents, cleaners, hair dye, bleach, nail polish, etc.?*
    b. *Probe: For <u>auto</u> sector shops: What about solvents, cleaners, paints, etc.?*
    c. *Probe: Do you worry about inhaling chemicals or getting them on your skin?*
5. What do you do to ensure the safety of your employees?

6. How do you learn about working with chemicals/products?
    a. *Probe: Do you feel like you have sufficient knowledge about potential hazards and how to work with these chemicals safely?*
    b. *Probe: Do you feel like your workers have sufficient knowledge about potential hazards and how to work with these chemicals safely?*
7. How do your workers get trained to work with chemicals/products safely?
    a. *Probe: When are they trained: At hire, periodically, annually, etc.?*
    b. *Probe: Who trains them?*
    c. *Probe: Has training changed their behavior about health and safety?*
8. What would make it easier to improve worker safety or reduce chemical use in your workplace?
    a. *Probe: Such as lower cost for safer chemicals, fewer worker complaints of smelly chemicals, fewer customer complaints of smelly chemicals, fewer worker sick days, grants/loans for better equipment, the right thing to do, etc.?*
    b. *Probe: Challenges?*
9. How does the health of your employees affect your business? (i.e. productivity)
    a. *Probe: Do you provide paid sick time?*
    b. *Probe: Do employees come to work when they're sick?*
10. Do workers ever come to you with concerns about their safety or health at work?
11. What type, if any, of health insurance or assistance with health insurance does your business provide to employees?
    a. *Probe: If no insurance, ask about other types of insurance.*
